# Smartphone-based retrospective analysis for malaria hotspot detection

**DOI:** 10.31744/einstein_journal/2025AO1826

**Published:** 2025-11-24

**Authors:** Bernardo Maia da Silva, Jeevan Giddaluru, Lucas Esteves Cardozo, Felipe de Mello Martins, Alinne de Paula Rodrigues Antolini, Daniel Youssef Bargieri, Marcus Vinicius Guimarães de Lacerda, Wuelton Marcelo Monteiro, Vanderson de Souza Sampaio, Helder I Nakaya

**Affiliations:** 1 Universidade do Estado do Amazonas Graduate Program in Tropical Medicine Manaus AM Brazil Graduate Program in Tropical Medicine, Universidade do Estado do Amazonas, Manaus, AM, Brazil.; 2 Universidade de São Paulo Faculdade de Ciências Farmacêuticas Department of Clinical and Toxicological Analyses São Paulo SP Brazil Department of Clinical and Toxicological Analyses, Faculdade de Ciências Farmacêuticas, Universidade de São Paulo, São Paulo, SP, Brazil.; 3 Secretaria Municipal de Saúde de Manaus Environmental Management Manaus AM Brazil Environmental Management, Secretaria Municipal de Saúde de Manaus, Manaus, AM, Brazil.; 4 Universidade de São Paulo Instituto de Ciências Biomédicas Department of Parasitology São Paulo SP Brazil Department of Parasitology, Instituto de Ciências Biomédicas, Universidade de São Paulo, São Paulo, SP, Brazil.; 5 Fundação de Medicina Tropical Dr. Heitor Vieira Dourado Manaus AM Brazil Fundação de Medicina Tropical Dr. Heitor Vieira Dourado, Manaus, AM, Brazil.; 6 Instituto de Pesquisa Leônidas e Maria Deane Fiocruz Amazonas Manaus AM Brazil Instituto de Pesquisa Leônidas e Maria Deane, Fiocruz Amazonas, Manaus, AM, Brazil.; 7 Universidade Federal do Amazonas Graduate Program in Health Sciences Manaus AM Brazil Graduate Program in Health Sciences, Universidade Federal do Amazonas, Manaus, AM, Brazil.; 8 Fundação de Vigilância em Saúde do Amazonas Manaus AM Brazil Fundação de Vigilância em Saúde do Amazonas, Manaus, AM, Brazil.; 9 Institut Pasteur de São Paulo São Paulo SP Brazil Institut Pasteur de São Paulo, São Paulo, SP, Brazil.; 10 Hospital Israelita Albert Einstein São Paulo SP Brazil Hospital Israelita Albert Einstein, São Paulo, SP, Brazil.

**Keywords:** Malaria, Smartphones, GPS, SiPoS, Spatial analysis

## Abstract

**Objective:**

To leverage passively collected retrospective smartphone location data to accurately identify malaria transmission hotspots through the development of a novel tool, the Sickness Positioning System.

**Methods:**

We collected anonymized location history data from over 200 malaria-infected individuals using Google Takeout. Sickness Positioning System applies density-based clustering techniques to this passively collected data to identify areas of elevated infection risk. The identified hotspots were subsequently validated through field investigations.

**Results:**

Sickness Positioning System successfully pinpointed multiple areas of increased malaria transmission risk. Field validation confirmed two previously unrecognized mosquito breeding sites as active sources of transmission. When compared to Brazil's federal surveillance system – which updates data annually – Sickness Positioning System provided a more precise and timely spatial assessment of malaria spread.

**Conclusion:**

Sickness Positioning System demonstrates a cost-effective, objective, and timely method for identifying malaria transmission hotspots. This approach has the potential to significantly enhance disease surveillance and facilitate more targeted public health interventions.

## In Brief

Silva et al. developed the Sickness Positioning System, which uses retrospective smartphone GPS data from malaria patients to identify transmission hotspots. Field validation confirmed mosquito breeding sites, showing that Sickness Positioning System can enhance malaria surveillance and guide targeted interventions.

## Highlights

■ Patients gave informed consent to share GPS history from their smartphones.
■ Anonymized GPS data revealed stay points and malaria infection hotspots in Manaus.
■ Hotspots guided health agents to mosquito breeding sites, aiding malaria control.

## Graphical abstract

**Figure:**
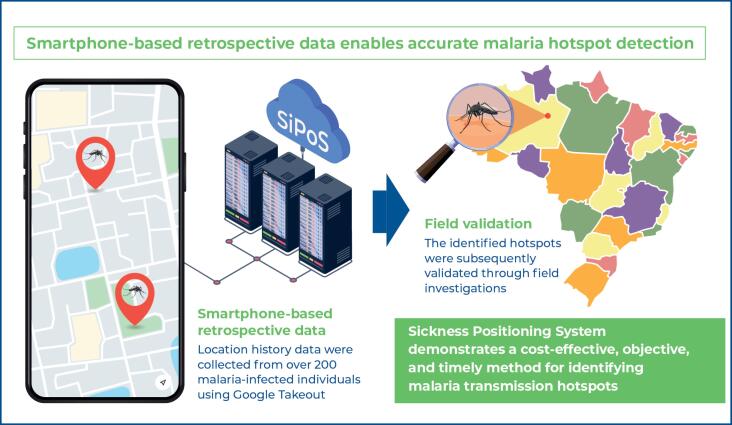

## INTRODUCTION

Malaria remains a significant global health challenge,^(1)^ and accurate identification of transmission hotspots is essential for effective control and elimination.^(2)^ Traditional methods for hotspot identification – such as Reactive Active Case Detection (RACD) – rely on patient recall and outdated information, often resulting in incomplete or imprecise data that limit the effectiveness of targeted interventions.

Recent advances in mobile technology have provided new opportunities to monitor population movements objectively. Many studies on digital epidemiology, particularly for pathogens like SARS-CoV-2,^(3,4)^ have utilized aggregated mobile phone data or real-time location information (*e.g*., from dedicated apps or call detail records) to evaluate the impact of social distancing measures and to model mobility patterns. However, none of these studies have specifically employed retrospectively collected smartphone location data obtained via Google Takeout, combined with density-based clustering techniques, to detect transmission hotspots.

In our study, we developed the Sickness Positioning System (SiPoS) to analyze anonymized historical location data passively collected from over 200 malaria-infected individuals. By leveraging this cost-effective and objective approach, we are able to pinpoint areas of increased infection risk without requiring users to download dedicated applications or carry extra devices. Field validation confirmed the presence of two previously unrecognized mosquito breeding sites as active transmission sources.

This methodological approach is innovative because it addresses an important gap in the digital epidemiology literature. While other studies have focused on real-time mobility metrics or aggregated operator data for COVID-19, our study is the first to use retrospective Google Location History data with density-based clustering to identify transmission hotspots. This demonstrates the versatility and potential of passively collected smartphone data in enhancing our understanding of malaria transmission dynamics and in supporting targeted public health interventions.

## OBJECTIVE

The goals of this study were to develop and evaluate the Sickness Positioning System, a novel digital epidemiology tool designed to leverage passively collected retrospective smartphone location data for the identification of malaria transmission hotspots. Specifically, we aimed to analyze anonymized Google Location History files from malaria-infected individuals, apply spatial clustering techniques to detect areas of elevated infection risk, and validate the identified hotspots through field investigations.

## METHODS

### Data collection

The data were obtained by the *Fundação de Medicina Tropical Dr. Heitor Vieira Dourado* (FMT-HVD) in Manaus. Patients presenting malaria symptoms were referred to the screening process, which is already routinely performed at the hospital. During this procedure, blood samples were collected and sent for diagnostic examination. Malaria-positive patients were informed and invited to participate in the current research. Patients who agreed to participate in the study were directed to a private room containing a computer, where the collaborator assisted them in sending the data through the SiPoS platform. Upon receiving the GPS data file from a patient, the SiPoS platform automatically processes and analyzes the patient's location history data (discussed in the following sections).

### Temporal classification and filtering

Each recorded GPS point is classified based on the time relative to the day of diagnosis. The temporal segmentation was classified into three periods: ‘symptomatic’, if the point was recorded within three days before sample collection; ‘exposure’, if the point is registered between 3 and 30 days before sample collection; ‘pre-exposure’, if the recorded point is between 60 and 90 days before collection. This classification is based on the mean time between the bite of the *Anopheles* mosquito bite and the onset of symptoms in malaria patients.^(5)^ The raw GPS data often comes with an accuracy error value for each recorded GPS point. For example, an accuracy error of 30 meters means that the recorded GPS coordinate has a geographic radius error rate of 30 meters. GPS points with accuracy error greater more than 50 meters were excluded from further analysis.

### Stay point identification

**Figure 1:**
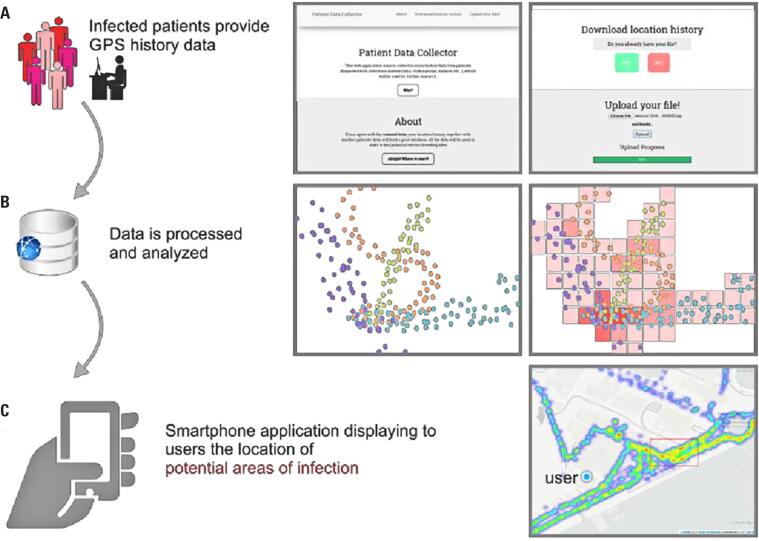
Overview of the project. (A) Location history data collection using the SiPoS platform from infected patients recruited at hospitals during routine procedures (B) GPS data processing and analysis, such as stay point identification and clustering analysis. (C) Viewing the potential transmission sites in real-time on SiPoS Web explorer

We assumed that a person has to stay in a specific location for some time to be infected by the mosquito carrying the malaria parasite. This step is necessary to identify and filter out the GPS points captured during motion in the city's main passageways. We used an algorithm to identify the "stay points" of the patients.^(6)^ The parameters, a spatial radius of 50 meters and a time threshold of 15 minutes, were used to identify the stay points, *i.e*., the patient spends at least 15 minutes within 50 meters of the radius. It is important to note that these stay points include places that might have been visited multiple times, such as home and work (re-visitation). Such places were defined as unique stay points. Figure 1 shows the schematic representation of all these steps.

### Clustering of stay points

We applied the DBSCAN algorithm (Density-based spatial clustering of applications with noise) to find the clusters of stay points from different patients.^(7)^ Patients who have passed through nearby regions have points represented in similar spatial groups (*i.e*., clusters). We used parameters Eps (distance radius) of 0.5 km and *Minpts* (minimum number of patients) of 3 to run the algorithm on our data.

### Stay point visitation share

We calculated a visitation share measure for each stay point to ascertain how often the respective patient visited each stay point. The visitation share of a stay point is defined as the number of times the stay point was visited divided by the total number of stay points visited during the period by a particular patient. The measure ranges from 0 to 1. A high value indicates high visitation by the patient to the stay point.

### Ethical aspects

Since our study involves collection of confidential information such as the user location history data, it raises several ethical questions. Therefore, before collecting data, our project was submitted and approved by the Ethics Committee of the *Fundação De Medicina Tropical "Doutor Heitor Vieira Dourado* through the Brazil Platform (CAAE: 68428917.0.0000.0005; # 2.135.257).

## RESULTS

### SiPoS: Sickness Positioning System

To obtain the geographic coordinates of the locations visited by the infected subjects, we developed a tool called SiPoS. This tool collects smartphones’ GPS location history through Google Takeout^®^. This service (found at https://www.google.com/settings/takeout) allows users to download various personal information stored on Google^®^ servers. The data is confidential and can only be accessed and made available by the users.

The SiPoS platform obtains no personal information from the participants. The users themselves have to access the online platform, and upon accepting the agreement displayed in the consent form, the platform guides them through the data collection process. To ensure privacy, each individual receives a unique code from our collaborator at the hospital. The participant uses this unique code to submit the data on the platform. The platform obtains the data anonymously *i.e*., it does not store the name, email address, or any other information regarding the identity of the infected person. Patients under treatment at the health centers are required to physically sign an additional consent form provided by the health centers and previously approved by local and national ethics committees.

After sending the GPS data through the SiPoS platform, the user receives an automatic confirmatory e-mail containing the consent form and instructions for withdrawing from the project if the participant wishes to. The GPS file containing the data is processed and stored in our secure server. The only information linked to the location data file is the unique code provided by the user when submitting the data. Our collaborators send the patient's diagnosis and additional information separately using the respective unique code.

**Figure 2:**
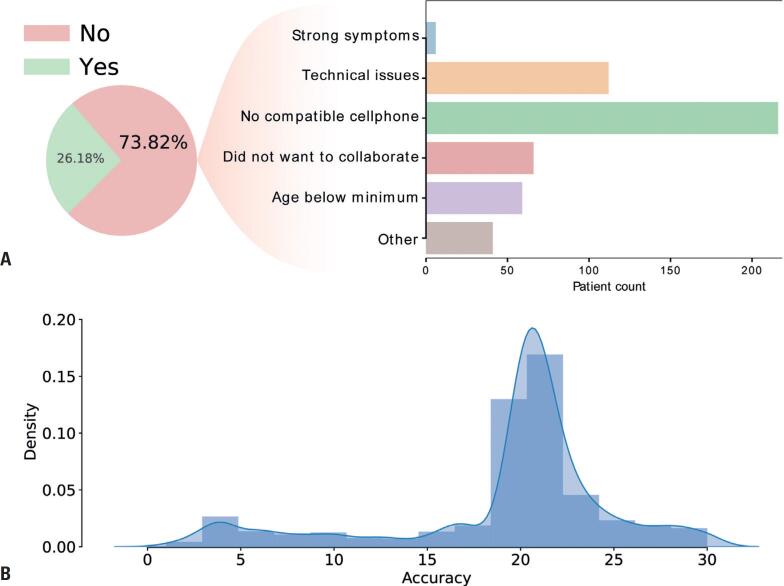
GPS data description of malaria-infected patients. (A) Recruitment of patients in the study and the reasons for not participating. (B) Distribution of accuracy error values of the retrieved GPS data. Most of the GPS points had a higher density of accuracy error around 20 and 25 meters

The SiPoS online platform (https://sipos.fcf.usp.br/) provides patients with data submission instructions, registers their consent, and collects the GPS history data from their cell phones. Our team approached over eight hundred malaria-infected patients, and around two hundred agreed to participate in our study. A small fraction of patients refused to participate (a little over 6%), and most could not send us the data due to technical issues or incompatible cell phones (Figure 2A). A hundred and four patients had GPS location data available with good accuracy and enough retrospective GPS data for the analyses. We discarded the coordinate points with an accuracy error value of more than 50 meters from the analyses (refer to the methods section). After filtering, most GPS points had an accuracy error measure of 20 meters (Figure 2B).

**Figure 3:**
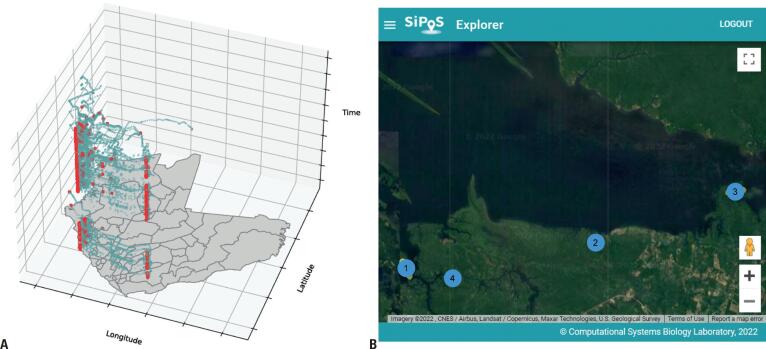
Potential hotspot clusters of Mosquito-Human Malaria Transmission. (A) 2D (left) and 3D (right) maps show patient FMT-112 stay and moving points. (B) Stay points of all the patients identified inside Manaus. Potential hotspot clusters detected inside the Tarumã region of Manaus (zoomed region). NA points represent stay points that were not assigned a cluster label by the DB-SCAN algorithm

For further processing, we used only the exposure and symptomatic periods data, *i.e*., data up to 30 days before the day of diagnosis (Figure 1S, Supplementary Material). We then identified places visited by each patient in the period mentioned above. Figure 3A represents 2D and 3D geographic plots of the stay points (places visited by the patient) and moving points of the patient FMT-112. The columnar formation of red dots on the 3D plot above the map indicates meaningful stay point locations for the patient. For this patient, in particular, it is possible to observe two columns of red dots representing the places of residence and work. We restricted the stay points used in our analysis to points recorded only during the night (between 5 pm and 6 am) because female anopheline mosquitoes have blood-feeding activity in the evening.^(8)^ Only about 25% of all the identified stay points were unique locations, and the rest were recurrently visited. Upon identification of the stay points of all patients, we clustered the stay points (refer to methods) recorded inside the Manaus region. Patients who have visited nearby places represented the same spatial groups (*i.e*., clusters). These clusters represent potential transmission hotspots, the locations more frequently visited by several diagnosed patients. We detected 56 stay point clusters, out of which most stay points were found in the urban areas (southwest). We discarded the main avenues of Manaus city as transmission hotspots (since everyone had a stay point around these locations). We also found several potential hotspot clusters outside the urban area of Manaus. Figure 3B shows the stay locations visited by all the patients in Manaus, and the zoomed area shows the potential stay point clusters in the Tarumã region.

We used the SiPoS explorer dashboard to visualize these stay points clusters from all the patients. These hotspot clusters were then overlapped and compared with the "likely zone of infection" derived from Brazil's Epidemiological Surveillance Information System for Malaria (SIVEP). SIVEP is, so far, the Brazilian gold-standard database for malaria surveillance. Our comparison revealed that most of our hotspots were previously reported in SIVEP, but some were new. Most importantly, because of operational issues, there is a delay between data gathering in the field and data entering in SIVEP databases, which causes a lag in information availability. On the other hand, our tool provided information on the likely site of infection in real time. We also observed that 97% of the stay points identified were within a distance of 1 km from a SIVEP hotspot, and 100% of points were within 2 kms distance. This covers almost the entirety of the area surface of the city (data not shown).

**Figure 4:**
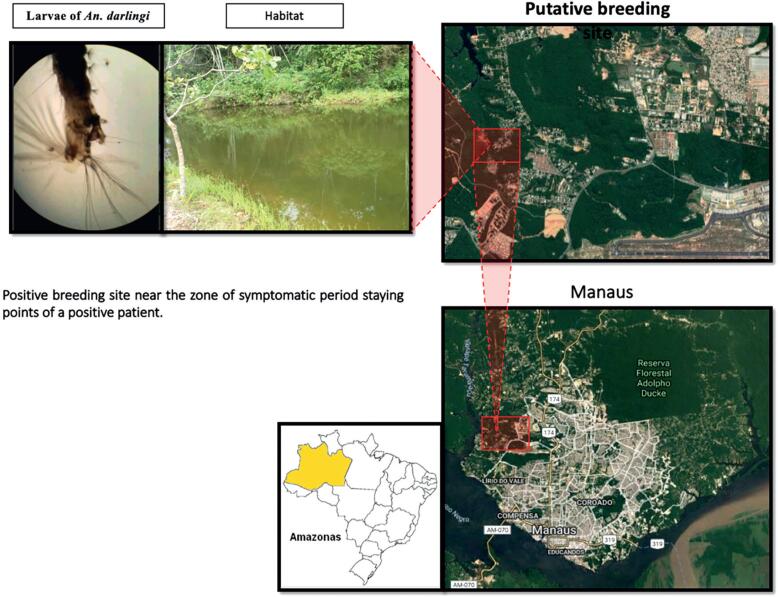
Field validation of transmission hotspots. Manaus map showing patients’ stay points and SIVEP hotspots, including locations visited by the field agents for validation of hotspots A, B, and C. After sending a team to hotspot C, a location with a stale body of water was determined, with ideal conditions for the development of Anopheles larvae. The collection of water samples showed the presence of larvae. A photo of the head of an *A. darlingi* larvae on a 10x microscope is displayed

After exploring these potential hotspot clusters on the SiPoS explorer dashboard, we sent medical entomology specialists to investigate putative sites and identify *Anopheles* breeding sites to validate these likely transmission hotspots. Among the operational issues, the difficulty in accessing access to the location due to private property was the most reported. Despite the difficulty, our team identified malaria vector larvae in two visited putative hotspots. Figure 4 shows stay points and SIVEP hotspots plotted on the Manaus map, including the locations visited for field validation by the entomology specialists. The field validation of hotspot A, B and C shows stay points visited by the patients and the potential transmission zones surveyed near the stay points. Potential hotspot C includes one of the identified putative hotspots.

In addition, we noticed that the stay points in the zone of hotspot C had the lowest visitation share values (0.001, 0.002, and 0.1666), indicating that the patients visited these locations only once or rarely during the whole exposure period and potentially got infected at these locations. All these findings validate our approach and methods, as they allow the identification of reproductive foci in an endemic region and effective control of the mosquito and, consequently, the disease.

## DISCUSSION

Historically, malaria control and elimination involved the detection of clinical, parasitological, and serological transmission markers in people to help identify transmission pockets and, thus, direct hotspot interventions. However, recent interest has focused on evaluating the transmission using innovative technologies.

We successfully employed our SiPoS platform to be used by the patients and health officials to submit the location and clinical data. A secure server was used to retrieve and store the data. We implemented an algorithm that identified locations visited by the patients where they possibly could have been infected by a mosquito. Performing clustering analysis of these locations allowed us to pinpoint the transmission hotspots. The tool showed applicability in a real-world epidemiological issue, such as accurately recognizing the places of infection transmission, which validates the usability of GPS data from mobile phones in disease surveillance. With the contribution of location data from more patients, we can identify additional transmission hotspots and achieve efficient disease control, thus effectively reducing disease in high-incidence regions and contributing to advances in Malaria elimination.

Due to the project's innovative aspect, we faced several challenges for which no reference was available to guide us. To our knowledge, no study on malaria has used retrospective GPS cell phone data for epidemic surveillance. The first challenge is the patient acceptance in providing the cell phone GPS history data. We believe that one primary (unspoken) concern is related to privacy. To solve this issue, we ensured that our system would not acquire personal information of the participants. Apart from that, only a quarter of the interviewed patients could provide their data (Figure 2A). The reasons include the lack of a smartphone equipped with GPS and other technical issues (for example, the user did not remember their google password for authentication). We also did interviews on high-profile social media channels to publicize the project among the population. Furthermore, we noticed that patients were more likely to participate in the project when the interviewer was wearing a lab coat.

Next, we had to define the data filtering methods and tune the parameters used in the analyses. For instance, some cell phones provide GPS data with low accuracy because the patients might frequently turn off their GPS on their cell phones (probably to save battery), leading the Google algorithm to estimate the location coordinates from nearby Wi-Fi devices. Other reasons include multipathing or satellite geometry errors leading to inaccuracies. Our challenges included but were not limited to, determining the use of moving and stay points: 1. To determine the use of moving and stay points in our study context; 2. Adjust the number of patients in a cluster to be considered a hotspot; 3. Optimal segmentation of the location history of individuals into "pre-exposure", "exposure" and "symptomatic"; 4. Whether the "pre-exposition" data can be used as a background control (*i.e*. places frequently visited by several patients, such as shopping malls, parks, and the city downtown, that are not likely to be a hotspot for transmission). To solve such issues, we discussed our strategies with specialists from different fields: epidemiologists, mathematicians, computer scientists, and malaria experts.

Most cases of mosquito-human malaria transmission occur in non-urban areas. In this study region, we assumed that patients probably got infected in rural areas or near the forest areas. The challenge was pinpointing the transmission locations in a large and "irregular" area. One approach we could use to address this issue is to restrict our search for places previously defined by SIVEP as "likely sites of infection".

## CONCLUSION AND FUTURE APPLICATION

One application we have not foreseen is to use retrospective GPS data to determine if an individual's malaria episode is due to reinfection or recurrence of symptomatic Malaria. To implement this, we will select the patients that had several episodes of malaria in the past six months and check whether they visited or not these potential hotspots of infection. These hotspots can be either from SIVEP or the hotspots we determined located outside Manaus.

Finally, we propose that the SiPoS platform could be applied to investigate various infectious diseases, not only Malaria. Each disease has its characteristics, and the challenge is thoroughly establishing them thoroughly. We are currently applying SiPoS to investigate diseases transmitted by mosquitoes adapted to rural and urban areas, such as *Aedes aegypti* (dengue, chikungunya, yellow fever, and Zika) and sandflies (leishmaniasis). Additionally, we will use SiPoS to study human-to-human contagious diseases such as Measles and Tuberculosis.
