# Supplementary material for: Smartphone-based retrospective analysis for malaria hotspot detection
Source: Einstein (Sao Paulo). 2025 Nov 24;23:eAO1826. doi: 10.31744/einstein_journal/2025AO1826 (PMC12671644; doi:10.31744/einstein_journal/2025AO1826)
Supplement: Supplementary file 1 [file 2317-6385-eins-23-eAO1826-suppl1.pdf]

SUPPLEMENTARY MATERIAL

Smartphone-based retrospective analysis for malaria hotspot detection

Bernardo Maia da Silva, Jeevan Giddaluru, Lucas Esteves Cardozo, Felipe de Mello Martins, Alinne de Paula Rodrigues Antolini, Daniel Youssef Bargieri, Marcus Vinicius Guimarães de Lacerda, Wuelton Marcelo Monteiro, Vanderson de Souza Sampaio, Helder I Nakaya

DOI: 10.31744/einstein_journal/2025AO1826

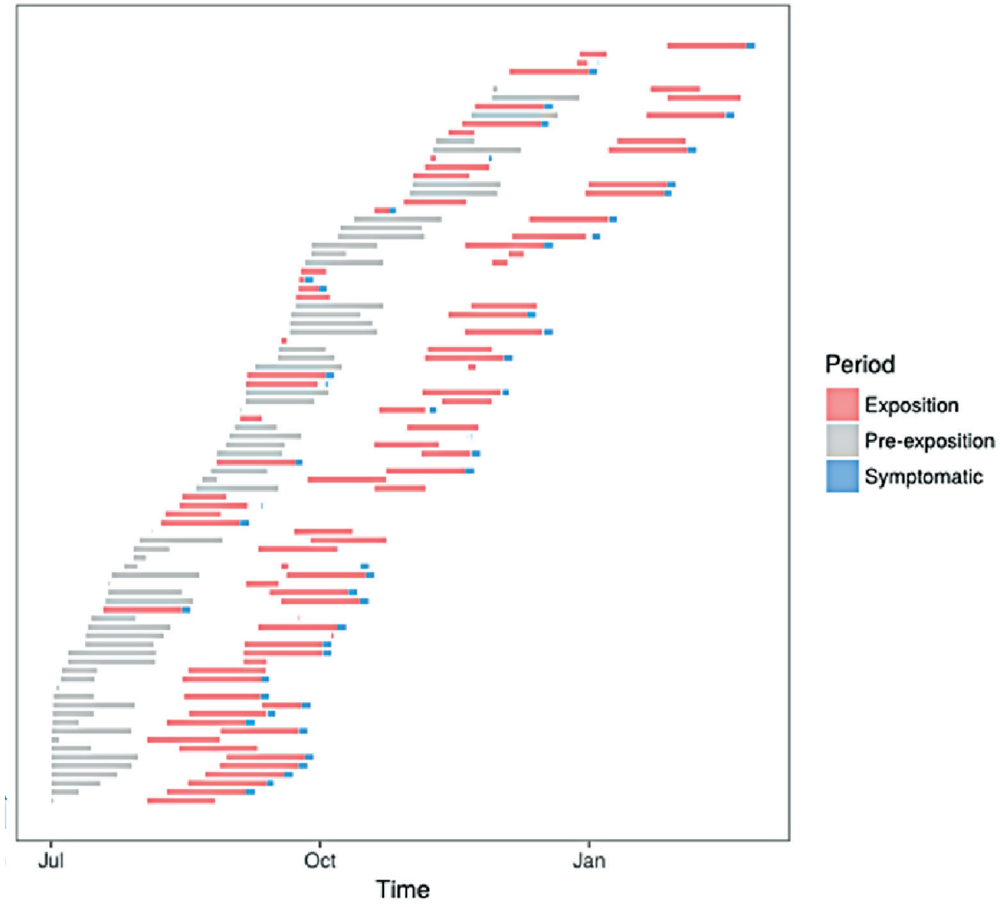

Figure 1S. Chart showing pre-expostion, exposition and symptomatic periods for each patient present in the study. Each line represents a patient. The x axis represents the months of the year
